# De-Escalation Strategies of (Chemo)Radiation for Head-and-Neck Squamous Cell Cancers—HPV and Beyond

**DOI:** 10.3390/cancers13092204

**Published:** 2021-05-04

**Authors:** Alexander Rühle, Anca-Ligia Grosu, Nils H. Nicolay

**Affiliations:** 1 Department of Radiation Oncology, University of Freiburg—Medical Center, Robert-Koch-Str. 3, 79106 Freiburg, Germany; alexander.ruehle@uniklinik-freiburg.de (A.R.); anca.grosu@uniklinik-freiburg.de (A.-L.G.); 2 German Cancer Consortium (DKTK) Partner Site Freiburg, German Cancer Research Center (dkfz), Neuenheimer Feld 280, 69120 Heidelberg, Germany

**Keywords:** head-and-neck cancer, head-and-neck squamous cell carcinoma, oropharyngeal cancer, HPV, radiotherapy, chemotherapy, cisplatin, cetuximab, de-escalation

## Abstract

**Simple Summary:**

HPV-related oropharyngeal squamous cell carcinoma patients have a very good prognosis but are often suffering from long-term treatment-induced toxicities. Therefore, a plethora of de-escalation trials is examining whether treatment for HPV-induced oropharyngeal carcinoma can be de-escalated without compromising the favorable outcomes. The purpose of this review was to present and critically discuss the published as well as the ongoing de-escalation trials in head-and-neck squamous cell carcinoma, in particular for HPV-related oropharyngeal carcinoma. De-escalation studies are using several approaches such as radiotherapy dose reduction, target volume reduction, omission of concomitant chemotherapy, replacement of cisplatin through less toxic systemic agents, omission of adjuvant (chemo)radiation after primary surgery and selection of suitable patients by induction chemotherapy or peritherapeutic hypoxia imaging. Although many promising results have been obtained from several Phase I and II trials, the two Phase III de-escalation trials failed to show the equivalence of the de-escalated treatment arm, so that so far, no treatment de-escalation can be recommended outside of clinical trials.

**Abstract:**

Oncological outcomes for head-and-neck squamous cell carcinoma (HNSCC) patients are still unsatisfactory, especially for advanced tumor stages. Besides the moderate survival rates, the prevalence of severe treatment-induced normal tissue toxicities is high after multimodal cancer treatments, both causing significant morbidity and decreasing quality of life of surviving patients. Therefore, risk-adapted and individualized treatment approaches are urgently needed for HNSCC patients to optimize the therapeutic gain. It has been a well-known fact that especially HPV-positive oropharyngeal squamous cell carcinoma (OSCC) patients exhibit an excellent prognosis and may therefore be subject to overtreatment, resulting in long-term treatment-related toxicities. Regarding the superior prognosis of HPV-positive OSCC patients, treatment de-escalation strategies are currently investigated in several clinical trials, and HPV-positive OSCC may potentially serve as a model for treatment de-escalation also for other types of HNSCC. We performed a literature search for both published and ongoing clinical trials and critically discussed the presented concepts and results. Radiotherapy dose or volume reduction, omission or modification of concomitant chemotherapy, and usage of induction chemotherapy are common treatment de-escalation strategies that are pursued in clinical trials for biologically selected subgroups of HNSCC patients. While promising data have been reported from various Phase II trials, evidence from Phase III de-escalation trials is either lacking or has failed to demonstrate comparable outcomes for de-escalated treatments. Therefore, further data and a refinement of biological HNSCC stratification are required before deescalated radiation treatments can be recommended outside of clinical trials.

## 1. Introduction

With more than 600,000 new cases and more than 350,000 deaths per year, head-and-neck squamous cell carcinoma (HNSCC) is a common malignancy globally and causes considerable rates of morbidity and mortality. While tobacco and alcohol consumption remain key risk factors for the majority of cases, chronic infection with human papillomavirus (HPV) is an emerging cause for HNSCC development, especially for oropharyngeal squamous cell carcinoma (OSCC). Various studies have demonstrated the superior prognosis of patients with HPV-driven OSCC, leading to a high proportion of long-term survivors that may be especially affected by long-term treatment-related toxicities [1,2,3]. Considering the very good prognosis of this patient cohort on the one side and the burden of chronic high-grade toxicities caused by cancer treatment, a plethora of Phase I-III trials are now investigating de-escalation strategies for HPV-associated OSCC. Besides strategies for this distinct biological subgroup of patients, treatment de-escalation approaches have also been proposed for non-HPV-associated HNSCCs.

In this review, we outline different de-escalation strategies for HNSCC patients that are currently pursued in clinical trials and summarize the existing evidence with a particular focus on HPV-positive OSCC.

## 2. Methods

A literature search with the search terms head-and-neck neoplasms/cancer, head-and-neck squamous cell cancer/carcinoma, oropharyngeal neoplasms/cancer/carcinoma, HPV/human papillomavirus, radiotherapy de-escalation and radiotherapy de-intensification in several combinations was performed using MEDLINE and Web of Science. In addition, current clinical trials examining de-escalation strategies for (HPV-positive) head-and-neck squamous cell carcinomas were searched using ClinicalTrials.gov. Both original research papers and conference abstracts were included in this narrative review.

## 3. De-Escalation Strategies for HPV-Positive Oropharyngeal Cancers

### 3.1. Dose De-Escalation for Definitive Chemoradiation

As there is profound evidence of dose-response relationships for normal tissues in the head-and-neck area, treatment dose de-escalation for patient subgroups with favorable prognoses has been subject to intensive investigation in various clinical trials [4,5,6]. At least retrospective analyses of the National Cancer Database including 759 patients with HPV-positive OPSCC did observe similar oncological outcomes of dose de-escalated radiotherapy compared to standard radiotherapy after balancing for other prognostic parameters [7].

In the initial single-arm trial by Chera and colleagues, 44 HPV-positive OSCC patients with a minimal smoking history received definitive chemoradiation with a reduced radiotherapy dose (60 Gy) and a less toxic cisplatin dose (30 mg/m^2^ weekly) [8,9] (Table 1).

After a median follow-up of 36 months, the three-year local control (LC), distant metastasis-free survival (DMFS) and overall survival (OS) still ranged at 100%, 100% and 95%, respectively. These findings were followed by another Phase II trial with 140 patients that received the same chemoradiation de-escalation without the need for mandatory post-treatment biopsies and neck dissection, which were replaced by a [^18^F] FDG-PET/CT at 10–16 weeks after completion of chemoradiation [5]. In this trial, the 2-year loco-regional control (LRC), DMFS and OS amounted to 95%, 91% and 95%, respectively. Although this single-arm trial still lacked a control group, the results were comparable to historical cohorts and therefore show the potential of this de-escalation approach. In a further follow-up study, the Phase II LCCC 1612 trial (NCT03077243), the investigators currently examine whether this moderate treatment de-escalation approach can also safely be pursued in distinct patients with HPV-positive OSCC and a relevant smoking history (>10 pack years). In this context, it has been suggested that the p53 wild-type status may be a surrogate parameter to determine the viral induction of OSCC in smokers. Therefore, the LCCC 1612 trial investigates dose de-escalation for patients with HPV-associated OSCC and either minimal smoking history (≤10 pack years) or relevant smoking history but wild-type p53 status.

In contrast to the single-arm trial designs of Chera and colleagues, the NRG-HN002 Phase II trial enrolled 306 patients with locally advanced HPV-related OSCC and no smoking history in a randomized fashion that compared radiation-dose de-intensified chemoradiation (60 Gy with 40 mg/m^2^ cisplatin weekly in 6 weeks) with mildly accelerated radiotherapy alone (60 Gy in 5 weeks) [6]. It should be emphasized that both trial arms deviated from the standard treatment dose of 70 Gy and can therefore be considered as de-escalation arms. While patients in the chemoradiation group exhibited a progression-free survival (PFS) of 90.5% after two years, accelerated radiotherapy alone resulted in a 2-year PFS of 87.6%, therefore failing to meet the predetermined PFS acceptability criterion [10]. Both trial arms passed the predetermined toxicity benchmark, namely the swallowing-related quality of life (QoL) measured by the MD Anderson Dysphagia Inventory (MDADI). The results of this study suggest that omission of concomitant chemotherapy compromises the outcomes of HPV-positive OSCC patients in the context of radiation dose de-escalation.

The PacCis study, a multi-center randomized Phase III trial, compared dose de-escalated chemoradiation with a total dose of 63.6 Gy and concomitant paclitaxel plus cisplatin versus standard chemoradiation with a total dose of 70.6 Gy and concomitant 5-FU plus cisplatin for HNSCC patients irrespectively of their HPV status [14]. The disease-free survival after 3 years in the subgroup of HPV-positive OSCC patients amounted to 84.6% for the experimental arm and 83.9% for the standard arm, while the 3-year OS was 92.3% in the dose de-escalated paclitaxel/cisplatin group and 83.5% in the standard 5-FU/cisplatin group. However, the gastrostomy tube dependence after 12 months was comparable between the experimental (10.5%) and the standard arm (9.5%). The number of p16-positive OSCC patients was too low (*n* = 49) to draw definitive conclusions; however, moderate dose de-escalation accompanied by taxane-based chemotherapy may be a de-escalation strategy that warrants further investigations.

### 3.2. Dose De-Escalation for Adjuvant Radiation TREATMENTS

The MC1273 trial employed a Phase II design which included 80 patients with adequately resected HPV-positive OSCCs and at least one risk factor (≥T3, ≥N2 (according to the 7th TNM Edition), extranodal extension [ENE], lymphovascular or perineural invasion), and only patients with a smoking history of below 10 pack years were eligible for enrolment [11]. Trial patients received adjuvant chemoradiation with a considerable de-escalation of radiation doses to 30 Gy (or 36 Gy in case of ECE). Radiotherapy was applied twice daily (either 1.5 Gy or 1.8 Gy depending on ECE), therefore shortening the adjuvant treatment time to only 2 weeks. Docetaxel 15 mg/m^2^ was administered concomitantly on days 1 and 8 during radiotherapy. The concept of aggressive dose de-escalation in the adjuvant treatment situation still resulted in excellent oncological outcomes, displayed by the 2-year LRC, PFS and OS of 96.2, 91.1% and 98.7%, respectively. However, it should be recognized that concomitant adjuvant chemotherapy is commonly only applied for either incomplete resection or ECE [15,16,17]; therefore, simultaneous administration of docetaxel in the MC1273 trial may have constituted a treatment escalation regarding systemic therapy for the majority of included patients without the respective risk factors [18]. However, both the QoL and the swallowing function were shown to be significantly improved at 1 year after completion of radiotherapy compared to the assessment prior to radiotherapy.

The DART-HPV trial (NCT02908477) is the follow-up Phase III trial of the MC1273 trial and randomly assigns patients after resection of HPV-positive OSCCs, irrespectively of the smoking history, to either the 2-week chemoradiation regimen as employed in the MC1273 study or to standard (chemo)radiation with 60 Gy. The primary endpoint will be the 2-year adverse event rate.

A risk-adapted de-escalation approach depending on pathological risk factors after primary transoral resection was used in the ECOG 3311 Phase II study with 519 enrolled patients. In this trial, patients with a low risk for locoregional recurrence (T1-2, N0-1 according to the 7th TNM edition) did not receive adjuvant radiotherapy, whereas patients with an intermediate risk profile (clear/close margins, ECE <1 mm, 2–4 metastatic lymph nodes, perineural invasion, lymphovascular invasion) were randomized between adjuvant radiotherapy with 50 Gy or 60 Gy. Patients in the high-risk group (positive resection margin, >1 mm ECE or ≥5 lymphonodal metastases) were treated with cisplatin-based chemoradiation to a dose of 66 Gy. Recruitment of this study has been completed, and first results were published at the ASCO 2020 conference [12]. The 2-year LRC amounted to 93.9% (low-risk group without adjuvant radiotherapy), 95.0% (intermediate-risk group with de-escalated adjuvant radiotherapy of 50 Gy), 95.9% (intermediate-risk group with standard adjuvant radiotherapy of 60 Gy) and 90.5% (high-risk group with cisplatin-based chemoradiation of 66 Gy). Grade 3/4 treatment-related adverse event rates were reported to be 15%/2% in the surgery only group, 13%/2% in the de-escalated radiotherapy group and 25%/0% in the standard radiotherapy group. Based on these Phase II trial data, the authors suggest that omission of postoperative radiotherapy is appropriate for patients of the low-risk group and that de-escalated radiotherapy with 50 Gy and without chemotherapy is sufficient for patients of the intermediate-risk group, meaning uninvolved surgical margins, <5 involved lymph nodes and minimal (<1 mm) ECE after transoral resection. A Phase III follow-up trial is currently in preparation that compares the de-escalation approach of the ECOG 3311 trial with optimal non-surgical treatment.

The currently recruiting non-randomized DELPHI trial (NCT03396718) also investigates postoperative radiation dose de-escalation depending on the risk profile. In the first de-escalation level, patients with an intermediate-risk profile (≤pT3, negative resection margins, maximum 3 involved lymph nodes, no ECE) will receive de-escalated radiotherapy with a 10% dose reduction (i.e., 54 Gy for the tumor bed, 45 Gy for the cervical lymphatics), whereas patients with at least one postoperative high-risk feature (R1 status, pT4, ≥4 positive lymph nodes, ECE) will undergo chemoradiation, also with a 10% dose reduction (i.e., 59.4 Gy for the tumor bed, 45 Gy for cervical lymph nodes). If 10% dose reduction is proven to be a safe concept (defined as maximal 3 tumor recurrences in the first 2 years for the first 30 patients), a second de-escalation step will follow. Here, radiotherapy dose will be reduced to 48.4 Gy (tumor bed)/39.6 Gy (cervical lymphatics) in the intermediate-risk group and 55 Gy (tumor bed)/39.6 Gy (cervical lymphatics) in the high-risk group.

While the previously outlined trials aimed to de-escalate treatment by reducing treatment dose, the AVOID trial investigated the feasibility of omitting the adjuvant radiotherapy altogether to the site of the primary tumor after transoral resection and selective neck dissection [13]. In this single-arm trial, 60 patients received adjuvant radiotherapy to the cervical lymphatics (60 Gy for involved sites, 54 Gy for non-involved sites), whereby radiation treatment to the resected primary site was omitted. The 2-year LC and 2-year OS amounted to 98.3% and 100%, respectively. Although a control group was missing, treatment-related toxicities were relatively low compared to historical cohorts; for instance, no patients required gastrostomy tube placement during follow-up. The median dose to the resected primary site ranged at 39.6 Gy, which may be sufficient for targeting residual HPV-positive tumor cells. It should be noted that only patients with small (T1-2) primary tumors and absence of risk factors (resection margin ≥2 mm, no perineural or lymphovascular invasion) were included in this trial, thereby representing a highly pre-selected patient group.

A combination of both treatment volume and radiotherapy dose de-escalation in the adjuvant situation is currently investigated in a single-arm Phase II trial (NCT03729518) performed at the University of Pennsylvania. Patients with HPV-related pT0-3, pN0-2b, M0 (7th TNM edition) OSSCs with <5 positive lymph nodes (i.e., pN1 according to the 8th TNM edition) after transoral resection and ipsilateral neck dissection are eligible for this study. The ipsilateral high-risk neck region is treated with 50 Gy instead of 60 Gy, and the treatment of the contralateral neck is de-escalated both by lowering the radiotherapy dose (45 Gy) and by reducing the target volume. Concomitant chemotherapy is applied in patients with positive resection margins and ECE, whereby chemotherapy may be omitted for microscopic ECE (≤1 mm). The 2-year LRC will be analyzed as the primary endpoint of the study.

### 3.3. Definitive (Chemo) Radiation Versus Surgery Plus Adjuvant (Chemo) Radiation

Regarding the choice of optimal treatment for HPV-positive OSCC, the results of the ORATOR trial provide the first data from a randomized comparison between transoral resection plus neck dissection (including postoperative (chemo)radiation depending on pathology) and definitive (chemo)radiation for T1-2, N0-2, M0 (7th TNM edition) OSCCs: while the oncological outcomes were comparable between both groups, the primary endpoint, the 1-year swallowing-related QoL as measured by the MD Anderson Dysphagia Inventory, was significantly superior in the radiotherapy group, although a clinically meaningful difference, defined as a 10-point difference, was not reached (86.9 versus 80.1 points). The secondary endpoints, global QoL and emotional QoL, were both significantly and clinically meaningfully superior in the radiotherapy group. The percentage of patients receiving total oral diet without any restrictions at 1 year after treatment amounted to 100% in the radiotherapy group and only 84% in the surgery group (*p* = 0.055). Even though a small number of non-HPV-positive OSCC patients were included, almost 90% of patients exhibited p16-positive tumors as surrogate parameter for patients’ HPV status. Based on this trial, the ORATOR II trial focusing on treatment de-escalation for HPV-positive OSCC patients (NCT03210103) is currently recruiting [19]: 140 patients with HPV-positive T1-2, N0-2 (8th TNM edition) OSCC will be randomized between either de-escalated definitive radiotherapy with 60 Gy (plus concomitant chemotherapy depending on nodal status) or transoral surgery followed by de-escalated adjuvant radiotherapy with 50–60 Gy depending on pathological risk factors. OS as compared to historical cohorts is the primary endpoint of this study, and the authors hypothesize that the 2-year OS will be greater than 85%. Secondary end points include 1-year swallowing-related QoL, other patient-reported QoL parameters, treatment-related toxicity rates and feeding tube rate at 1 year. Besides reducing the radiotherapy dose, the ORATOR II trial also investigates chemotherapy de-escalation: While a weekly cisplatin regimen (40 mg/m^2^) is used in the definitive treatment cohort in case of metastatic lymph node involvement, no concomitant chemotherapy is applied after resection, even for positive resection margins or ECE. Furthermore, other currently recruiting randomized trials aim to compare definitive radiotherapy with primary surgery in early-stage OSCC such as the ‘best of’ study of the EORTC (NCT02984410) or the QoLATI trial of the DAHANCA group (NCT04124198).

### 3.4. Omission of Adjuvant Chemoradiation in Case of Incomplete Resection or ECE

The significance of the addition of chemotherapy to adjuvant radiotherapy was established by two Phase III landmark trials, and adjuvant chemoradiation is commonly applied for incompletely resected HNSCCs or in case of ECE [15,16,17]. The omission of concurrent chemotherapy for HPV-positive OSCC in the adjuvant setting is currently the subject of the Phase II/III PATHOS trial. The PATHOS study enrolls patients with HPV-related OSCCs (T1-3, N0-2b) and a negative smoking history, in whom the choice of adjuvant treatment depends on pathological risk factors after resection [20]. Patients with intermediate risk factors such as lymph node metastases, lymphovascular or perineural invasion or close resection margins are randomized between adjuvant dose-deescalated radiotherapy (50 Gy) or standard adjuvant radiotherapy (60 Gy). In case of high-risk factors (incomplete resection or ECE), patients are randomized between postoperative radiotherapy and postoperative chemoradiation with 60 Gy [16]. The trial is currently recruiting, and results are awaited in 2027.

The ADEPT trial (NCT01687413) is a randomized Phase III trial that compares adjuvant chemoradiation with radiotherapy alone for HPV-related OSCC patients in case of ECE after primary resection. Unfortunately, this trial closed due to poor accrual and funding issues [21].

### 3.5. Induction Chemotherapy for Selection of Patients Suitable for De-Escalation

Patient selection for radiation dose or volume de-escalation strategies based on their response to induction chemotherapy has been recently tested in several Phase II trials (Table 2).

In the ECOG 1308 trial, 80 patients with HPV-associated OSCCs received induction bio-chemotherapy with cisplatin, paclitaxel and cetuximab [22]. Patients with a complete clinical response after induction chemotherapy were treated with dose-deescalated radiotherapy (54 Gy) and concomitant cetuximab, whereas patients without a complete clinical response received cetuximab-based bio-radiotherapy to a total dose of 70 Gy. The 2-year PFS for patients with complete clinical remission (70% of all patients) and de-escalated treatment amounted to 80%. Interestingly, the 2-year PFS of patients with a smoking history of 10 pack years or less and a complete clinical response amounted to 96%, showing the relevance of smoking as a key prognosticator for HPV-positive OSCC patients. Only 15% of the patients in the de-escalated treatment arm exhibited higher-grade dysphagia, compared to 29% in the standard treatment arm.

In a further Phase II study of Chen and colleagues, treatment de-escalation was performed based on the response to induction chemotherapy [23]. Patients with complete or partial remission received paclitaxel-based chemoradiation with 54 Gy, while the remaining patients were treated using a total dose of 60 Gy, which still constituted a de-escalation. In this trial, the 2-year PFS amounted to 92% for both groups, while the prevalence of gastrostomy tube dependence at 3 and 6 months after treatment was only 2% and 0%, respectively.

The Quarterback study was a small trial in which 20 patients with HPV-positive OSCC (Stage III–IV without distant metastases according to the 7th TNM edition) and a smoking history of ≤20 pack years received induction chemotherapy (docetaxel, carboplatin, 5-FU) followed by standard chemoradiation (70 Gy) or de-escalated chemoradiation (54 Gy) depending on the response to induction treatment [24]. While the 3-year PFS was 87.5% for the standard treatment group, it was only non-significantly reduced in the dose-deescalated cohort with 83.3% (*p* = 0.85).

A rather complex study design was used in the OPTIMA trial: 62 HPV-positive OSCC patients (T1-4, N2-3 according to the 7th TNM edition) received induction chemotherapy consisting of carboplatin and nab-paclitaxel [25]. Patients in the low-risk group (≤T3, ≤N2b, ≤10 pack years) were allocated to different treatments based on their response to induction chemotherapy: patients with >50% radiological response (measured by RECIST v1.1 assessment) received radiotherapy only to a dose of 50 Gy, patients with 30–50% radiological response were treated with dose de-escalated chemoradiation (45 Gy plus concomitant paclitaxel, 5-FU and hydroxyurea), and patients with <30% radiological response underwent high-dose chemoradiation (75 Gy plus concomitant paclitaxel, 5-FU and hydroxyurea). Patients belonging to the high-risk group (T4, >N2c, >10 pack years) were treated with de-escalated chemoradiation (45 Gy plus concomitant paclitaxel, 5-FU and hydroxyurea) in the case of ≥50% response or high-dose chemoradiation (75 Gy plus concomitant paclitaxel, 5-FU and hydroxyurea) in the case of lesser response. While the 2-year PFS was above 90% for all treatment arms, toxicity was strongly reduced in the de-escalated treatment groups. For instance, 82% of patients in the high-dose chemoradiation arm (75 Gy) received a gastrostomy tube compared to 0% in the de-escalated group (radiotherapy with 50 Gy). The results of this trial seem even more favorable, considering that not only low-risk HPV-associated OSCC patients were included but also patients with advanced tumors (T4 or >N2c) and/or a smoking history exceeding 10 pack years.

### 3.6. Treatment De-Escalation in Dependence of the Peri-Therapeutic Tumor Hypoxia Dynamics

The stratification of HNSCC treatment based on molecular or imaging biomarkers seems promising, and hypoxia dynamics have been reported to predict patient outcomes: while persistent tumor-associated hypoxia during chemoradiation has been shown to significantly deteriorate the LRC of HNSCC patients, patients with early-resolving tumor hypoxia exhibit a better prognosis [26,27,28,29]. Hypoxia can be non-invasively monitored by functional PET imaging using hypoxia-specific radiotracers such as [^18^F]fluoromisonidazole (FMISO) or [^18^F]fluoroazomycin arabinoside (FAZA) [29,30,31].

Based on the predictive role of peritherapeutic hypoxia dynamics, Lee and colleagues used the FMISO imaging in week 1 of chemoradiation to select HPV-positive OSCC patients suitable for treatment de-escalation (Table 3). In this study, 30% of included patients exhibited an early tumor hypoxia response during chemoradiation, and therefore, received de-escalated treatment for the metastatic lymph nodes to a total dose of 60 Gy in the definitive setting [27]. After a median follow-up of 32 months, both 2-year LRC and 2-year OS were 100%.

Very recently, the same group reported about their results of the 30 ROC trial in which an aggressive FMISO-based de-escalation approach was applied for patients with T1-2, N1-2b (7th TNM edition) HPV-related OSCCs (*n* = 16) or cervical HPV-positive cancers of known primary (CUP) (*n* = 3) undergoing primary resection without an a priori neck dissection [32]. Patients with absent tumor hypoxia prior to radiotherapy or resolution of tumor hypoxia within the first two weeks of chemoradiation received a considerably de-escalated chemoradiation regimen with 30 Gy, whereas patients with persistent tumor hypoxia were treated with standard chemoradiation of 70 Gy. Fifteen of the 19 included patients exhibited either no tumor hypoxia at baseline (*n* = 6) or early resolution during treatment (*n* = 9), as quantified by FMISO PET/CT imaging, and therefore received a considerably de-escalated chemoradiation regimen. Of these 15 patients, 11 patients were found to have a pathological complete response in the mandatory neck dissection at 4 months after radiotherapy (whereby 2 patients only exhibited minimal tumor cells with unclear viability). After a median follow-up period of 34 months, 2-year LRC was 94.4% for the entire cohort, and there were no radiotherapy-related higher-grade toxicities in the de-escalation group. Although the results of this personalized hypoxia-directed treatment de-escalation approach are promising, it remains unanswered to what extent the mandatory neck dissection after chemoradiation may have improved the outcome of this cohort. The authors therefore aim to validate their findings in a larger non-randomized Phase II trial (NCT03323463) with 300 participants in which no neck dissection after de-escalated chemoradiation (30 Gy in 15 fractions) is conducted in HPV-positive and hypoxia negative T1-2, N1-2c (7th TNM edition) OSCC patients.

Other biomarkers, e.g., pertaining to the immune microenvironment, may also provide biology-directed treatment de-escalation, but necessary trials are lacking so far [33,34,35,36,37].

### 3.7. Replacement of Cisplatin with Other Systematic Agents

The MACH-NC meta-analysis has established cisplatin monotherapy as the treatment of choice for concomitant chemoradiation of HNSCCs [38]. On the other side, combining radiotherapy with the EGFR antibody cetuximab was shown to be superior to radiotherapy alone in a Phase III study by Bonner and colleagues [39]. Based on the favorable toxicity profile demonstrated in this study, two large Phase III trials aimed to show the non-inferiority and significant reductions in treatment-related toxicities by replacing cisplatin with cetuximab in combination with radiotherapy for HPV-positive OSCCs (Table 4) [40,41].

The De-ESCALaTE-HPV study compared cetuximab-based bio-radiotherapy (70 Gy in 35 fractions) versus cisplatin-based chemoradiation for OSCC patients (T3-4, N0 or T1-4, N+ and <10 pack years). The rate of higher-grade toxicities after 2 years as the primary endpoint of this study and the global QoL were comparable between both groups. However, the bio-radiotherapy group exhibited a significantly lower 2-year OS (89.4% versus 97.5%) [40]. Even when analyzing only patients of the lowest-risk group, the inferior outcome of bio-radiotherapy remained significant and clinically relevant (5.2% difference in 2-year OS favoring the cisplatin group) [42]. In line with the significantly worse OS, patients in the cetuximab group had a three-times higher risk for local recurrences than patients in the cisplatin group (16.1% versus 6.0%).

The RTOG 1016 study had a similar design and randomized 849 patients with HPV-positive OSCC (T1–2, N2a–3 or T3–4, N0–3 according to the 7th TNM edition) between cisplatin-based chemoradiation and cetuximab-based bio-radiotherapy with 70 Gy [41]. OS was defined as the primary endpoint in the RTOG 1016 study, and the cetuximab cohort exhibited a 5-year OS of 77.9%, which was almost 10% lower than the control group in which a cisplatin-based chemoradiation was applied (84.6%). Similar to the results of the De-ESCALaTE-HPV trial, patients in the cetuximab group had a significantly higher risk for locoregional relapse in comparison with patients in the cisplatin-cohort (17.3% versus 9.9% after 5 years). Again, there was no difference in terms of the prevalence of higher-grade toxicities between both arms. Interestingly, there was a comparable OS between the cetuximab (5-year OS 84.0%) and cisplatin group (5-year OS 84.6%) in patients with very good performance status (Zubrod status of 0); however, this result should be interpreted with caution, as it was an unplanned post-hoc analysis [42].

These trials provide the first head-to-head comparison between concomitant cisplatin and cetuximab for HNSCC radiation treatment and furthermore provide the first negative Phase III evidence for treatment de-escalation of HPV-positive OSCC patients. It remains debatable if the results may be extrapolated to HPV-negative HNSCCs.

Given the negative attempts to replace cisplatin by cetuximab, other trials currently test whether immunotherapy such as the PD-1 inhibitor nivolumab may replace cisplatin as radiotherapy combination partner for HPV-associated OSCCs. Increased PD-L1 levels have been described in HPV-positive OSCCs, and palliative immunotherapy has been found to result in superior outcomes for HPV-positive OSCCs than for HPV-negative HNSCCs, giving a rationale to test radioimmunotherapy as de-escalation approach [43,44]. At the moment, the ongoing NRG-HN005 Phase II/III trial (NCT03952585) compares three treatment groups: standard cisplatin-based chemoradiation with 70 Gy, cisplatin-based dose-deescalated chemoradiation with 60 Gy and nivolumab-based radioimmunotherapy with 60 Gy for patients with early-stage (T1-3, N0-1, M0, 8th TNM edition) HPV-related OSCC and minimal smoking history (≤10 pack years). Another single-arm Phase II trial of the MD Anderson Cancer Center (NCT03799445) is currently investigating the combination of the CTLA-4 antibody ipilimumab and the PD-1 antibody nivolumab in combination with dose de-escalated radiotherapy of 60 Gy for patients with HPV-associated OSCC. In a Canadian and European multi-center trial (NCT03410615), standard cisplatin-chemoradiation is currently compared with durvalumab-based radioimmunotherapy for locoregionally advanced intermediate-risk HPV-positive OSCC, whereby the radioimmunotherapy group is further randomized between durvalumab or durvalumab/tremelimumab maintenance. While the JAVELIN head-and-neck 100 Phase III trial failed to show a benefit of adding avelumab to definitive chemoradiation for locally advanced HNSCC [45], it will be interesting to see the safety and efficacy of checkpoint inhibitor-based radioimmunotherapy in these de-escalation studies for HPV-related OSCCs.

## 4. Beyond HPV: De-Escalation Strategies for HNSCCs

### 4.1. Technical Radiotherapy Approaches

Modern radiation techniques such as intensity-modulated radiotherapy (IMRT) with lower dose restrictions to critical normal tissues, e.g., parotid glands or mandibular bone, have resulted in considerably lower toxicity rates such as xerostomia and osteoradionecrosis compared to conventional 3D radiation techniques [46,47,48]. However, radiotherapy-related dysphagia remains a frequent and major toxicity, severely affecting the long-term quality of life of surviving HNSCC patients [49,50]. A plethora of studies have shown dose-volume relationships between the prevalence of late dysphagia and radiation dose to distinct anatomical structures such as the pharyngeal constrictor muscles, masseter muscle, larynx, oral cavity, cervical esophagus and soft palate [51]. Very recently, Nutting et al. reported the results of the randomized Phase III DARS trial comparing dysphagia-optimized IMRT with standard IMRT in 112 patients with either oro- or hypopharyngeal carcinoma [52,53]. Dysphagia-optimized IMRT aimed to spare the pharyngeal constrictor muscles outside of the high-dose treatment volumes [53]. Consecutively, the mean dose to the inferior pharyngeal constrictor amounted to 49.8 Gy in the standard arm versus 28.4 Gy in the experimental arm, while the superior/middle pharyngeal constrictor doses were 57.2 Gy in the standard arm compared to 49.7 Gy in the dysphagia-optimized IMRT arm. The primary endpoint, the swallowing-related quality of life measured by the MDADI at 1 year after radiotherapy, ranged at 77.7 in the standard arm, while it was 70.3 in the experimental arm (*p* = 0.016). Although showing promising results regarding the late dysphagia rate in patients treated by dysphagia-optimized IMRT, the relatively low patient number for a Phase III trial (*n* = 112) makes definitive conclusions difficult. However, dose reductions for non-tumor-involved swallowing-related structures is a promising strategy and has the potential to reduce treatment-related dysphagia rates [54].

Another approach to lower treatment-induced toxicities may be a reduction of the CTV-PTV margin to adjust for patient-related and technical inaccuracies that traditionally ranges at about 5 mm for head-and-neck cancer (Table 5).

Daily image-guided radiotherapy (IGRT) may allow to reduce this margin from 5 mm to 3 mm, as shown in previous studies [55,56,57]. Navran and coworkers reported significantly fewer levels of acute grade 3 mucositis and acute grade 3 dysphagia when CTV-PTV margins were reduced to 3 mm, while oncological outcomes were similar between both groups [55]. In another study, 367 patients were analyzed and compared depending on the CTV-PTV margin: the 2-year LRC for the 5 and 3 mm margin cohorts were 78% and 80%, respectively, whereas the gastrostomy tube dependence after 1 year amounted to 10% and 3%, respectively (*p* = 0.001), favoring reduced margins [56]. Van Kranen et al. calculated a dose reduction of approximately 1 Gy per mm margin reduction in organs-at-risk [58].

### 4.2. De-Escalation in the Post-Operative Situation

While the overall good prognosis of HPV-positive OSCCs makes these cancers attractive for de-escalation of (chemo)radiation both in the definitive and adjuvant setting, several trials have also investigated de-escalation strategies for HNSCCs irrespective of the HPV status.

In a Phase II trial including 73 patients (both with HPV-positive and HPV-negative HNSCCs), Contreras et al. investigated whether postoperative radiotherapy to the pathologically negative neck can be safely omitted [59]. While about a quarter (24%) of patients received only postoperative radiotherapy to the primary site, the remaining patients also received ipsilateral neck treatment in case of unilateral nodal metastases. After a median follow-up of 53 months, only 2 patients experienced recurrence within the non-irradiated neck, whereby both patients also had a recurrent primary tumor. The omission of postoperative radiotherapy for the surgically staged non-metastatic cervical lymphatics resulted in a good preservation of patients’ QoL that was comparable to the baseline prior to radiotherapy. Vice versa, the randomized non-inferiority PET-NECK trial examined whether planned neck dissections may be omitted in case of PET-guided surveillance [60]. In this study, 564 HNSCC patients (of which 75% exhibited p16-positive OSCCs) with Stage N2 or N3 disease (7th TNM edition) were randomized between planned neck dissection either before or after definitive (chemo)radiation versus FDG-PET-based surveillance at 12 weeks after the end of chemoradiation. In the PET surveillance arm, neck dissection was only conducted, if the post-radiotherapeutic PET-CT showed incomplete or lack of response. As expected, PET imaging-guided surveillance led to fewer neck dissections than planned dissection surgery (54 versus 221), and prevalence of surgical complications were comparable between both arms (42% versus 38%). The 2-year OS amounted to 84.9% in the PET-surveillance group and 81.5% in the planned neck dissection group, indicating at least non-inferiority of the image-guided surveillance group (both in the p16-negative and p16-positive group). Given the comparable survival outcomes and QoL data of both arms, PET-guided surveillance with neck dissections only for absent or incomplete response should be preferred to planned dissection for HNSCC patients with Stage N2 disease, as it results in fewer operations and is more cost-effective. Considering the low number of Stage N3 disease (9 patients in the PET-surveillance arm), this approach warrants further investigation for this high-risk subgroup.

The DIREKHT trial (NCT02528955) is currently investigating a de-escalation approach in the postoperative situation for patients with pT1-3, pN0-N2b tumors of the oral cavity, oropharynx and larynx or pT1-2, pN1 tumors (7th TNM edition) of the hypopharynx who exhibit negative resection margins and at least one of the two criteria: (1) pT2, R ≥ 5 mm, L0 and Pn0 and/or (2) ≤3 ipsilateral lymph node metastases. A previous retrospective assessment could show an excellent 2-year OS (94.7%) and a very low 2-year locoregional recurrence rate (1.0%) of this patient population, suggesting that a de-escalation study may be justified for these patients [61]. Depending on the exact tumor stage, resection margin and surgery, patients are allocated to one of the three treatment arms: While patients in Arm A receive de-escalated radiotherapy of 56 Gy to the tumor bed, patients in Arm B are spared from contralateral neck irradiation but receive 64 Gy to the primary tumor region, whereby patients in Arm C receive dose de-escalation to the primary tumor region (56 Gy) and ipsilateral neck radiotherapy only. Treatment plan analyses of the first 30 patients demonstrated significantly decreased treatment volumes and considerable dose reductions of the constrictor muscles [62]. It will be interesting to observe how the primary endpoint of this study, the 2-year LRC, will be compared to historical cohorts.

### 4.3. Weekly Versus Three-Weekly Cisplatin

Modification or de-escalation of the concomitant cisplatin regimen has been tested as another approach to reduce chemoradiation-induced toxicities. Based on several large trials and meta-analyses, cisplatin is commonly applied to a dose of 100 mg/m^2^ in three cycles during radiotherapy (Days 1, 22 and 43) [63,64,65]. Noronha et al. compared this three-weekly application with a weekly cisplatin administration of 30 mg/m^2^ in a randomized non-inferiority trial including 300 HNSCC patients, whereby almost all patients (93%) were treated postoperatively and only a small minority (7%) in a definitive situation [66]. The LRC after 2 years was considerably lower in patients receiving weekly cisplatin compared to the standard treatment arm (58.5% versus 73.1%). On the other hand, the prevalence of higher-grade acute toxicities was reduced from 84.6% in the three-weekly cisplatin application schedule group to 71.6% in the weekly application group. It has to be noted that the median cumulative cisplatin doses varied massively between both trial arms and ranged at 300 mg/m^2^ in the standard and only 210 mg/m^2^ in the experimental arm. Additionally, the vast majority (87.3%) of patients exhibited oral cavity tumors, making the extrapolation of these results to other head-and-neck entities such as (HPV-positive) oropharyngeal or laryngeal cancer difficult.

Recently, Kiyota and colleagues reported preliminary results of the randomized Phase II/III JCOG1008 trial with 261 patients who were treated with postoperative chemoradiation [67]. Patients in the standard arm received 100 mg/m^2^ cisplatin every three weeks, while patients in the experimental arm were treated with weekly 40 mg/m^2^ cisplatin. The median cisplatin doses amounted to 280 mg/m^2^ and 239 mg/m^2^ in the three-weekly and weekly administration group, respectively. After a median follow-up of more than 2 years, 3-year OS was 59.1% in the standard arm and 71.6% in the experimental arm, clearly showing a non-inferiority of weekly cisplatin in the adjuvant setting. As expected, patients receiving weekly cisplatin suffered from significantly less treatment-related toxicities: for instance, higher-grade (≥grade 3) neutropenia occurred in 48.8% of patients in the standard treatment arm and in 35.3% in the experimental arm. Although the final publication has to be awaited, the results may define a new treatment standard for adjuvant chemoradiation in the future. As there is no subgroup analysis of the HPV-positive OSCC patients in the conference abstract, it will be interesting to compare the results of HPV-negative and HPV-positive HNSCC in terms of the cisplatin schedule in the final publication.

The RADIO trial, a prospective open-label randomized clinical trial (NCT03649048), is currently comparing low-dose weekly cisplatin 40 mg/m^2^ with high-dose three-weekly cisplatin 100 mg/m^2^ for locally advanced HNSCC, whereby p16 status is used as stratification parameter. Hearing-related QoL both in the general cohort and in the elderly as well as rates of ototoxicity are the primary endpoints of the study. The study is recruiting at the moment, and study completion is estimated for 2025.

Safe fractionation of high-dose cisplatin 100 mg/m^2^ using a protracted application of 25 mg/m^2^ over 4 days was investigated in the multi-center randomized Phase II GORTEC 2015-02 CisFRad trial [68]. A total of 124 patients, either receiving definitive or adjuvant chemoradiation, were randomized between single-application and fractionated high-dose cisplatin 100 mg/m^2^ q3w. While the oncological outcomes were comparable between both groups, patients receiving fractionated cisplatin were able to receive a significantly higher cumulative cisplatin dose (median 291 mg/m^2^ versus 280 mg/m^2^), which was the primary endpoint of the study. Furthermore, fractionated cisplatin administration resulted in significantly fewer acute toxicities than single-application administration (35% versus 65% grade 3–4 toxicities). The findings regarding fractionated cisplatin administration are promising; however, further data are necessary to determine an adequate cumulative cisplatin dose for definitive and adjuvant chemoradiation. To date, three concomitant cycles of high-dose cisplatin remain the standard treatment for definitive chemoradiation in patients with locally advanced HNSCC [69].

## 5. Conclusions

Many clinical trials are currently recruiting patients in order to examine treatment de-escalation strategies with the long-term goal of reducing treatment-associated toxicities without affecting the superior survival rates of HPV-positive OSCC patients. Selection of patients based on biological tumor features or response to induction chemotherapy showed promising results in Phase II trials. Additionally, several Phase II trials demonstrated good oncological results for adjuvant treatment de-escalation after surgical tumor treatment. However, the two negative Phase III cetuximab trials clearly demonstrate the importance of confirming all promising Phase II data compared to the current treatment standards. Based on the lack of positive Phase III evidence for treatment de-escalation in this context, no treatment de-escalation for HPV-related OSCCs can be currently recommended outside of clinical trials.

## Figures and Tables

**Table 1:** Overview of important (chemo)radiotherapy de-escalation trials in which the radiation dose/volume was de-escalated. CRT = chemoradiation, RT = radiotherapy, OS = overall survival, PFS = progression-free survival, LRC = local/locoregional control, DMFS = distant metastasis-free survival.

Study	# Patients	Phase	Study Arm(s)	Results
Chera et al. [8,9]	44	II	RT (60 Gy) + cisplatin (30 mg/m^2^ weekly)	3-year LRC 100% 3-year DMFS 100% 3-year OS 95%
NRG-HN002 [6,10]	306	II	RT (60 Gy) + cisplatin vs. RT (60 Gy)	2-year PFS 90.5% (RT + cisplatin) vs. 87.6% (RT) 2-year OS 96.7% (RT + cisplatin) vs. 97.3% (RT)
MC1273 [11]	80	II	Adjuvant RT (30 Gy in 1,5 Gy twice per day or 36 Gy in 1,8 Gy twice per day)	2-year LRC 96.2% 2-year PFS 91.1% 2-year OS 98.7%
ECOG 3311 (ASCO abstract [12])	519	II	Depending on the risk profile after resection: Regular aftercare (low-risk, group A), randomization between adjuvant RT with 50 Gy (group B) or 60 Gy (group C) (intermediate-risk), additive cisplatin-based CRT (66 Gy) (high-risk, group D)	2-year PFS Group A: 93.9% Group B: 95.0% Group C: 95.9% Group D: 90.5%
AVOID [13]	60	II	Omission of the postoperative RT for the primary tumor site	2-year LRC 98.3% 2-year PFS 92.1% 2-year OS 100%

**Table 2:** Summary of important (chemo) radiotherapy de-escalation trials in which induction chemotherapy was applied. RT = radiotherapy, OS = overall survival, PFS = progression-free survival, LRC = local/locoregional control, IC = induction chemotherapy, cCR = complete clinical remission, pCR = partial clinical remission.

Study	# Patients	Phase	Study Arm(s)	Results
ECOG 1308 [22]	80	II	In case of cCR after IC: RT (54 Gy) + cetuximab	2-year PFS 80% 2-year OS 94% For patients with cCR and 54 Gy-deescalated RT: 2-year PFS 96% 2-year OS 96%
Chen et al. [23]	44	II	After IC: RT (54 Gy) + paclitaxel for cCR or pCR, RT (70 Gy) + paclitaxel for absent cCR/pCR	2-year LRC 95% 2-year PFS 92%
Quarterback [24]	20	II	After IC: RT (70 Gy) + carboplatin vs. RT (56 Gy) + carboplatin	3-year PFS 87.5% (70 Gy) vs. 83.3% (56 Gy) 3-year OS 87.5% (70 Gy) vs. 83.3% (56 Gy)
OPTIMA [25]	62	II	Complex study conception and treatment arm allocation in dependence of response to IC	Entire cohort: 2-year LRC 98% 2-year PFS 94.5% 2-year OS 98%

**Table 3:** Summary of de-escalation trials based on assessment of peritherapeutic tumor hypoxia dynamics. RT = radiotherapy, CRT = chemoradiation, OS = overall survival, PFS = progression-free survival, LRC = locoregional control, DMFS = distant metastasis-free survival.

Study	# Patients	Phase	Study Arm(s)	Results
Lee et al. [27]	33	Pilot	De-escalation of RT dose (60 Gy instead of 70 Gy) for patients with early tumor hypoxia response in week 1 of CRT	Entire cohort: 2-year LRC 100% 2-year DMFS 97% 2-year OS 100%
Riaz et al. [32]	19	Pilot	De-escalation of RT dose (30 Gy instead of 70 Gy) for patients with absent tumor hypoxia at baseline or early resolution in the first 2 weeks of CRT	Entire cohort: 2-year LRC 94.4% 2-year PFS 89.5% 2-year OS 94.7%

**Table 4:** Phase III de-escalation trials aiming to replace cisplatin with cetuximab. RT = radiotherapy, OS = overall survival.

Study	# Patients	Phase	Study Arm(s)	Results
De-ESCALaTE-HPV [40]	334	III	RT (70 Gy) + cisplatin vs. RT (70 Gy) + cetuximab	2-year local recurrence rate 6.0% (RT + cisplatin) vs. 16.1% (RT + cetuximab) 2-year OS 97.5% (RT + cisplatin) vs. 89.4% (RT + cetuximab)
RTOG 1016 [41]	849	III	RT (70 Gy) + cisplatin vs. RT (70 Gy) + cetuximab	5-year PFS 78.4% (RT + cisplatin) vs. 67.3% (RT + cetuximab) 5-year local recurrence rate 9.9% (RT + cisplatin) vs. 17.3% (RT + cetuximab) 5-year OS 84.6% (RT + cisplatin) vs. 77.9% (RT + cetuximab)

**Table 5:** Retrospective comparisons analyzing different CTV-PTV margin concepts. CTV = clinical target volume, PTV = planning target volume, LRC = locoregional control, OS = overall survival.

Study	# Patients	Type	CTV-PTV Margin	Results
Navran et al. [55]	414	Retrospective	3 mm versus 5 mm	3 mm versus 5 mm: Overall acute grade 3 toxicity: 53.8% versus 65%, *p* = 0.032. Acute grade 3 mucositis: 30.8% versus 42.2%, *p* = 0.008 Acute feeding tube-dependence: 22.1% versus 33.5%, *p* = 0.026 Feeding tube-dependence after 3 months: 11.1% versus 20.4%, *p* = 0.012 2-year incidence of late grade ≥2 xerostomia: 15.8% versus 19.4%, *p* = 0.8 2-year LRC: 79.9% versus 79.2%, *p* = 1.0 2-year OS: 75.2% versus 75.1%, *p* = 0.9.
Chen et al. [56]	367	Retrospective	3 mm versus 5 mm	3 mm versus 5 mm: 3-year LRC: 80% versus 78%, *p* = 0.75 Feeding tube-dependence after 1 year: 3% versus 10%, *p* = 0.001 Incidence of posttreatment esophageal stricture: 7% versus 14%, *p* = 0.01

## Data Availability

Data sharing not applicable.
